# Loss of antiphospholipid antibody positivity post-thrombosis in SLE

**DOI:** 10.1136/lupus-2020-000423

**Published:** 2020-10-06

**Authors:** Muznay Khawaja, Laurence Magder, Daniel Goldman, Michelle A Petri

**Affiliations:** 1 Department of Medicine, Division of Rheumatology, Johns Hopkins University School of Medicine, Baltimore, Maryland, USA; 2 University of Maryland Medical Center, Baltimore, Maryland, USA; 3 Rheumatology, Johns Hopkins University, Baltimore, Maryland, USA

**Keywords:** antibodies, antiphospholipid, anticardiolipin, lupus erythematosus, systemic

## Abstract

**Background/Purpose:**

Loss of positivity of antiphospholipid antibodies has been observed in clinical practice post-thrombosis in patients with SLE with secondary antiphospholipid syndrome (APS). Our study defined the frequency of this loss and the duration before positivity recurred.

**Methods:**

In this prospective study, patients with SLE having at least two positive antiphospholipid markers prior to thrombosis and at least 1 year of follow-up after thrombosis were included. Antiphospholipid markers included lupus anticoagulant (dilute Russell viper venom test >45 s followed by mixing and confirmatory tests) and/or anticardiolipin titre (aCL IgG ≥20, aCL IgM ≥20 and/or aCL IgA ≥20). The percentage of visits with positive antiphospholipid markers after thrombosis was calculated. For patients with a negative antiphospholipid marker any time after thrombosis, survival estimates were performed to calculate the time to return of antiphospholipid positivity.

**Results:**

In APS due to SLE, complete loss of antiphospholipid positivity post-thrombosis was up to 41% for aCL IgG, 51% for IgM and 50% for IgA, but only 20% for those with lupus anticoagulant. Of those who at some point lost aCL IgG or became negative for lupus anticoagulant, the majority (60% and 76%, respectively) reacquired the antibody within 5 years. In contrast, of those who lost aCL IgM or IgA, fewer reacquired it within 5 years (37% and 17%, respectively).

**Conclusion:**

Intermittent positivity of antiphospholipid antibodies is present in APS due to SLE. These fluctuations make it difficult to decide on length of anticoagulation. Lupus anticoagulant is more likely to persist post-thrombosis.

## Introduction

Antiphospholipid syndrome (APS) can be a primary disorder or secondary to an autoimmune disease such as SLE.^1 2^ Thrombotic APS can present with arterial, venous or microvascular thrombosis in the setting of persistently positive antiphospholipid (aPL) antibodies.^3^ There are three types of aPL antibodies that can be measured in routine clinical practice. These include lupus anticoagulant, anticardiolipin (aCL) antibody and anti-beta-2 glycoprotein.^4^ The lupus anticoagulant is the most important test, with the strongest association with both thrombosis and pregnancy morbidity.^5–11^ It is tested through coagulation testing via a three-step process. The first step is a sensitive coagulation test such as dilute Russell viper venom test (dRVVT) or partial thromboplastin time. The next step is a mixing study. The final confirmatory test involves adding phospholipid, leading to, for example, the dRVVT confirm ratio.^12–14^ Multiple factors, not just the presence of the aPL antibody, play a role in the risk of future thrombosis.^15–22^ Lupus anticoagulant, high titre aCL antibodies, IgG isotype, persistence for 6 months or longer^23 24^ and multiple hits (such as oral contraceptive use or immobility) are all important.

The 1999 Sapporo classification criteria for APS included laboratory criteria of the lupus anticoagulant or medium to high titre aCL IgG or IgM positive over 6 weeks.^25^ The Sydney revision added a new requirement that the aPL antibody must be repeatedly positive over 3 months.^26–32^ Clinical criteria (thrombosis and pregnancy related) had to be present within 5 years of the positive aPL antibody assay. Only 59% of the patients meeting 1999 Sapporo classification criteria met the 2006 Sydney revision criteria.^27 28^

The Sydney classification criteria are difficult to apply to longitudinal data in SLE. In SLE, aPL antibodies behave like anti-double stranded DNA, in that there are fluctuations between positive and negative (and are unlike anti-Ro, La, RNP and Sm, which once positive, usually stay positive).

Loss of positivity of aPL antibodies has been observed in clinical practice post-thrombosis with secondary APS.^33^ There are no definite guidelines for optimal duration of treatment or discontinuation of anticoagulation in patients with APS who become negative. Our study defined the frequency of loss of aPL antibodies in SLE post-thrombosis, and determined the duration of time before positivity recurred.

## Methods

Our current study was based on prospective data collected as part of the Hopkins Lupus Cohort. All patients fulfilled revised American College of Rheumatology^34 35^ or Systemic Lupus International Collaborating Clinics^1^ classification criteria for SLE. Patients were seen every 3 months by protocol and aPL antibodies (lupus anticoagulant and aCL) were measured at every visit. Missed clinic visits were excluded. Lupus anticoagulant was measured by dRVVT as previously described followed by mixing studies and confirmatory testing when prolonged.^21^ aCL IgG, IgM and IGA were measured by ELISA (INOVA).

International Society of Thrombosis and Haemostasis (ISTH) guidelines were followed for lupus anticoagulant measurement. For RVVT confirm ratio calculation, standard Siemens testing kits were used. The package insert and our laboratory’s stated test performance on patients on oral anticoagulant therapy (vitamin K antagonists) was valid. Specificity studies were performed on known plasma samples. RVVT confirm was found to be positive in zero out of seven samples tested from patients known to be on oral anticoagulant therapy. In general, this testing strategy worked well as long as the international normalised ratio was not >4.0. Heparin levels up to 1 unit/mL had no effect due to the presence of a neutralising agent in both lupus anticoagulant screening reagent and lupus anticoagulant confirmation reagent.

Patients with SLE having at least two positive aPL antibody markers 3 months apart, prior to experiencing a thrombosis, were included. The starting point for all patients was entry in the cohort. Patients without at least 1 year of follow-up after the thrombosis were excluded. Positive aPL antibodies were defined as either dRVVT >45 s followed by a confirmatory test, or titres of aCL IgG, aCL IgM or aCL IgA exceeding 20. The cut-off value of 20 was used for aCL antibodies as values >20 have been associated with thrombosis in SLE^32^ and, as ‘medium’ titres, are part of the Sydney APS classification criteria.^36^ Anti-beta-2 glycoprotein antibodies were not included in this study as they were not measured at every visit. Arterial thrombosis was defined as: thrombotic stroke, myocardial infarction, other arterial thrombosis and digital gangrene. Venous thrombosis was defined as: deep venous thrombosis, pulmonary embolus or other venous thrombosis. Each event was confirmed by medical records. If a person had more than one thrombosis satisfying these conditions, we used the earlier one. Then, for each included patient, we examined their aPL results after the thrombosis.

### Statistical analyses

SAS 9.4 software was used for statistical analyses. The first analysis was to determine loss of aPL positivity after thrombosis. We measured the proportion of visits after the thrombotic event that were positive for an aPL antibody.

The second analysis was to determine the likelihood of return of positive aPL antibody (after becoming negative) at any time after thrombosis. Separate analyses were done for each aPL antibody. For each antibody, this analysis included those who were negative at some point in time after their thrombosis. Then, we used a Kaplan-Meier approach to estimate the risk of reacquisition of antibody over time since the visit with the negative aPL result. Those who never reacquired the antibody were censored.

The third analysis was to assess the determinants of aPL fluctuation. These determinants included baseline aCL titres (prethrombosis), baseline lupus anticoagulant ratio and double positivity (lupus anticoagulant+aCL).

## Results

### Analysis 1. Loss of aPL positivity after thrombosis

Analysis 1 was the determination of loss of aPL positivity after a thrombotic event (table 1). Among those with elevated aCL IgG prior to thrombosis, 41% lost the positive aCL IgG after thrombosis. Similarly, among those with elevated aCL IgM, 51% lost the positive IgM after thrombosis. In contrast, among those with prethrombosis lupus anticoagulant, only 20% of the patients were consistently negative after thrombosis.

**Table 1:** Characteristics from aPL-positive patients who experienced a thrombosis during cohort participation*

Patient characteristics	aCL IgG (n=34)	aCL IgM (n=35)	aCL IgA (n=8)	Lupus anticoagulant (n=35)
Age at time of thrombosis (years)				
<30	5 (15%)	3 (9%)	0 (0%)	7 (20%)
30–44	14 (41%)	11 (31%)	4 (50%)	13 (37%)
45–59	9 (26%)	16 (46%)	2 (25%)	10 (29%)
60+	6 (18%)	5 (14%)	2 (25%)	5 (14%)
Year at time of thrombosis				
2000–2009	22 (65%)	21 (60%)	5 (63%)	22 (63%)
2010–2018	12 (35%)	14 (40%)	3 (37%)	13 (37%)
Number of prethrombosis aPL measures				
2–4	3 (9%)	2 (6%)	1 (13%)	2 (6%)
5–9	6 (18%)	4 (11%)	1 (13%)	6 (17%)
10+	25 (74%)	29 (83%)	6 (75%)	27 (77%)
Proportion of prethrombosis aPL measures that were positive				
<25%	14 (41%)	17 (49%)	3 (38%)	9 (26%)
25%–49%	3 (9%)	7 (20%)	1 (13%)	8 (23%)
50%–74%	7 (21%)	4 (11%)	1 (13%)	7 (20%)
75%–99%	7 (21%)	4 (11%)	1 (13%)	4 (11%)
100%	3 (9%)	3 (9%)	2 (25%)	7 (20%)
Type of thrombosis				
Stroke	10 (29%)	8 (23%)	3 (38%)	8 (23%)
Myocardial infarction	3 (9%)	5 (14%)	2 (25%)	6 (17%)
Other arterial thrombosis	9 (26%)	9 (26%)	0 (0%)	8 (23%)
Digital gangrene	0 (0%)	2 (6%)	0 (0%)	1 (3%)
Deep vein thrombosis	11 (32%)	10 (29%)	2 (25%)	9 (26%)
Other venous thrombosis	1 (3%)	1 (3%)	1 (13%)	3 (9%)
Number of post-thrombosis aPL measures				
1–4	1 (3%)	3 (9%)	1 (13%)	4 (11%)
5–9	9 (26%)	4 (11%)	2 (25%)	9 (26%)
10+	24 (71%)	28 (80%)	5 (63%)	22 (63%)
Proportion of post-thrombosis aPL measures that were positive				
0%	14 (41%)	18 (51%)	4 (50%)	7 (20%)
<25%	6 (18%)	4 (11%)	0 (0%)	2 (6%)
25%–49%	5 (15%)	5 (14%)	0 (0%)	6 (17%)
50%–74%	4 (12%)	3 (9%)	1 (13%)	7 (20%)
75%+	3 (9%)	3 (9%)	1 (13%)	8 (23%)
100%	2 (6%)	2 (6%)	2 (25%)	5 (14%)

*This consists of those patients with at least two positive aPL tests before thrombosis. Some patients can appear in more than one column.

aCL, anticardiolipin; aPL, antiphospholipid.

### Analysis 2. Return of aPL after becoming negative after thrombosis

Analysis 2 was to determine return of aPL positivity after a period of negativity post-thrombosis. We considered those who were negative for aCL IgG any time after thrombosis, and determined the time until a positive aCL IgG titre recurred. There were 31 patients in the analysis. Of these, 14 (45%) never had a positive titre after thrombosis. Table 2a depicts the probability of reacquiring a positive aPL antibody after thrombosis. Similar calculations were performed for the other aPL tests. The great majority of patients lost their positivity during follow-up after the thrombosis. Those with the lupus anticoagulant usually (76%) regained it. Numerically, aCL IgA was the least likely to recur, followed by aCL IgM.

**Table 2a:** Estimated probability of reacquiring aPL antibody after a thrombosis among those who became negative after the thrombosis

aPL subtype	Number with thrombosis	Number negative after thrombosis	Number reacquiring a positive result	Estimated risk of reacquiring within 5 years (95% CI)
aCL IgG ≥20	33	31 (94%)	17 (55%)	60% (40% to 71%)
aCL IgM ≥20	35	33 (94%)	14 (42%)	37% (23% to 57%)
aCL IgA ≥20	8	6 (75%)	1 (12.5%)	17% (3% to 73%)
Confirmed lupus anticoagulant	35	30 (86%)	21 (70%)	76% (57% to 91%)
Prethrombosis positive for any of above	60	48 (80%)	34 (71%)	73% (59% to 86%)

**Table 2b:** Time from thrombosis to first negative aPL antibody

Time since thrombosis	IgG (n=31)	IgM (n=33)	IgA (n=6)	Lupus anticoagulant (n=30)	Any of the four aPLs (n=48)
<1 year	29 (94%)	31 (94%)	4 (67%)	19 (63%)	34 (71%)
1–3 years	2 (6%)	2 (6%)	1 (17%)	6 (20%)	7 (15%)
>3 years	0 (0%)	0 (0%)	1 (17%)	5 (17%)	7 (15%)

aCL, anticardiolipin; aPL, antiphospholipid.

Table 2b shows the timing of antibodies to become negative after thrombosis. For the majority the timing of becoming negative for an aPL antibody was within the first year after the thrombotic event. However, for the lupus anticoagulant, 37% of the time the dRVVT confirm did not become negative until 1 or more years after thrombosis.

**Figure 1:**
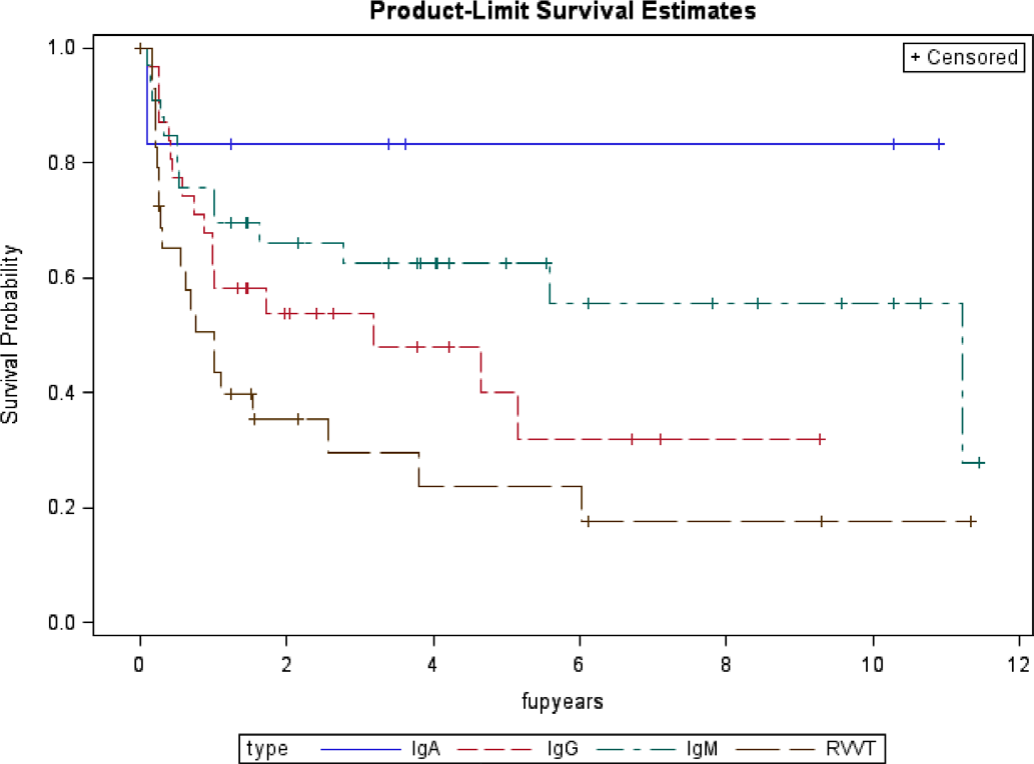
Probability of remaining antiphospholipid negative after thrombosis among patients with SLE who became negative after the thrombotic event. RVVT, Russell viper venom test.

Figure 1 depicts the probability of remaining aPL negative after thrombosis among patients with SLE who became negative after the thrombotic event. The two higher risk aPL antibodies, lupus anticoagulant (dRVVT confirm) and aCL IgG, are the most likely to become positive again.

### Analysis 3. Becoming antibody negative after thrombosis based on prethrombosis aPL levels

The third analysis was to examine the proportion of patients who lost aPL in the first year after thrombosis, based on prethrombosis characteristics. The results are shown in table 3.

**Table 3:** Proportion becoming antibody negative in the first year after thrombosis based on prethrombosis characteristics

Prethrombosis aPL status	Number	Number (%) negative in first-year post-thrombosis
aCL IgG >20	33	29 (88%)
aCL IgG >40	12	11 (92%)
aCL IgM >20	35	31 (89%)
aCL IgM >40	15	14 (93%)
aCL IgA >20	8	4 (50%)
aCL IgA >40	4	1 (25%)
Lupus anticoagulant	35	19 (54%)
Any of the above (aCL or lupus anticoagulant)	60	34 (57%)
IgG >20 but no other positive aPLs	9	9 (100%)
IgM >20 but no other positive aPLs	8	5 (63%)
IgA >20 but no other positive aPLs	2	1 (50%)
Any aCL >20 without lupus anticoagulant	26	20 (77%)
Lupus anticoagulant but no positive aCLs	7	5 (71%)
Lupus anticoagulant plus one or more aCLs >20	27	9 (33%)

aCL, anticardiolipin; aPL, antiphospholipid.

## Discussion

The presence of aPL antibodies (in particular, lupus anticoagulant) in patients with SLE is associated with thrombosis^37 38^ and increased mortality due to thrombotic events.^39^ High intensity warfarin was originally recommended as the treatment of choice after thrombosis in APS.^40–42^ However, usual intensity warfarin has been shown to be equally effective for prevention of recurrent thrombosis, with lesser bleeding risk.^43–45^ Most patients with APS will be anticoagulated long-term.

However, aPL antibodies can disappear post-thrombosis. Alarcon-Segovia found a decrease in aCL titres in four patients after a thrombotic event in patients with SLE and APS.^33^ Our study showed that loss of aPL positivity post-thrombosis occurred in 94% for aCL IgM, 94% for aCL IgG, 75% for aCL IgA and 86% for lupus anticoagulant (table 2a). If it occurred just at the time of thrombosis, it might reflect loss due to deposition in the thrombosis. More likely, it is simply the fact that aPL antibodies fluctuate over time. Use of prednisone can also be a potential explanation for aPL levels dropping after thrombosis.

Persistent aPL antibody in asymptomatic carriers is a risk factor for future thrombotic events.^46 47^ Persistence is defined as two positive titres (medium to high) in the APS classification criteria.^36^ This is why we designed our analysis to include only such patients with SLE. A definition of persistence, though, has not derived from longitudinal data. Our analysis (table 2a) found that 60% of aCL IgG-positive patients who were negative post-thrombosis developed a positive level again within 5 years, and 76% of lupus anticoagulant-positive patients who were negative post-thrombosis regained a positive value within 5 years. This contrasts with the results of a past study (Erkan *et al*) that found that aPL results remained stable three-quarters of the time on subsequent testing.^48^ The study by Erkan *et al*^48^ had a mean follow-up period of 2–3 years. However, Erkan *et al* selected patients based on high titre aCL IgG or lupus anticoagulant only. Our results, which apply only to SLE, do point out that aCL IgG and/or lupus anticoagulant are most likely to recur if they become negative post-thrombosis.

Thrombosis recurrence can occur despite the use of oral anticoagulants. Anticoagulation, though, significantly reduces the risk of thromboembolism in APS as shown by multiple studies.^49 50^ Martinez-Berriotxoa *et al*^51^ suggested that transiently positive aCL antibodies do not confer thrombotic risk. A systematic review showed that evidence linking the presence of aPL markers to subsequent thrombotic risk was low quality.^52^ Amory *et al* found that rates of death or re-thrombosis were not influenced by aPL results at baseline or follow-up.^53^ Our data show that recurrence of positive titres of aPL antibodies after initial negativity is common in APS due to SLE.

Currently, there are no definite guidelines for optimal duration of treatment in patients with secondary APS. The duration of anticoagulation after a thrombotic event varies, with studies showing a very high frequency of re-thrombosis in patients with SLE who stopped anticoagulation.^54 55^ There has been one case series of 44 patients with APS, in which oral anticoagulation was stopped. Comarmond *et al* showed that 54.5% of the 15 patients who had SLE had a re-thrombosis, but only 10% had a recurrent event in those who had negative aPL antibodies.^54^ Schulman *et al* showed prospectively that aPL-positive patients have a higher risk of thrombosis recurrence compared with aPL-negative patients when they stopped anticoagulation after 6 months (29% vs 14% over 4 years’ follow-up).^55^ Our results point out how difficult it is to define a patient with SLE as ‘positive’ or ‘negative’ for aPL markers, given the fluctuations over time. However, with lupus anticoagulant, we found less fluctuation over time.

For patients who have persistently negative aPL antibodies, there are no guidelines on discontinuation of anticoagulation. In contrast to the study by Schulman *et al*, a recent prospective study by Coloma Bazán *et al* showed that there were no new thrombotic episodes in patients with low-risk APS who developed persistently negative aPL antibodies when taken off anticoagulation.^56^ This study was done in 11 patients with primary APS, but the follow-up period was only 20 months. Our study disagrees, and implies that having negative aPL antibodies post-thrombosis in SLE is not sufficiently reassuring to stop anticoagulation. Thus, clinicians should be highly cautious before making the decision to stop anticoagulation in a patient with SLE with loss of aPL antibodies after a thrombotic event, as antiphospholipid antibodies are likely to recur. In addition, the decision to stop anticoagulation in these patients also depends on other risk factors for thrombosis, such as defined in the Hopkins Thrombosis Risk Equation^57^ and other risk equations.^58 59^ Our study results are in contrast to findings reported by Devignes *et al*,^60^ in which they reported extended persistence in aPL positivity in 89.6% of patients. It is unclear how many of them had thrombosis. Extended loss of aPL positivity post-thrombosis was up to 51% for aCL IgM in our study. Our study findings are similar to Devignes *et al*^60^ in that lupus anticoagulant tended to persist long term.

We acknowledge that our analyses are limited to a single centre. However, this is the only longitudinal study in which aPL antibodies were measured quarterly in all patients, removing the bias of short-term follow-up and selection bias. Another limitation is that anti-beta-2 glycoprotein antibodies were not included in these analyses. Thus, data regarding triple positive patients are not reported in this study.

## Conclusions

In APS due to SLE, complete loss of aPL positivity post-thrombosis occurred in up to 51% for aCL IgM and 20% for lupus anticoagulant. Sixty per cent of aCL IgG positive patients and 76% of lupus anticoagulant positive patients who were negative post-thrombosis developed a positive level again within 5 years. Lupus anticoagulant is more likely to persist post-thrombosis. Recognising that aPL antibody positivity may fluctuate in SLE will affect clinical decisions on continuation of anticoagulation.

10.1136/lupus-2020-000423.supp1 Supplementary data
