# Supplementary material for: Loss of antiphospholipid antibody positivity post-thrombosis in SLE
Source: Lupus Sci Med. 2020 Oct 6;7(1):e000423. doi: 10.1136/lupus-2020-000423 (PMC7539588; doi:10.1136/lupus-2020-000423)
Supplement: Supplementary data [file lupus-2020-000423supp001.pdf]

Analysis using a cutoff of aCL>40.

This is the analyses requiring a titer of 40 or more to be positive for aCL. The results are shown below in the next three tables. Note that the number of thromboses included becomes quite small.

Table 1 using the cutoff of 40: Characteristics from antiphospholipid positive patients who experienced a thrombosis during cohort participation[1].

Patient Characteristics	aCL IgG (n=12)	aCL IgM (n=15)	aCL IgA (n=4)	Lupus Anticoagulant (n=35)
Age at time of thrombosis				
<30	2 (17%)	1 (7%)	0	7 (20%)
30-44	3 (25%)	3 (20%)	1 (25%)	13 (37%)
45-59	4 (33%)	9 (60%)	2 (50%)	10 (29%)
60+	3 (25%)	2 (13%)	1 (25%)	5 (14%)
Year at time of thrombosis				
2000-2009	8 (67%)	11 (73%)	3 (75%)	22 (63%)
2010-2018	4 (33%)	4 (33%)	1 (25%)	13 (37%)
Number of pre-thrombosis aPL measures				
2-4	2 (17%)	1 (7%)	1 (25^)	2 (6%)
5-9	2 (15%)	1 (7%)	1 (25%)	6 (17%)
10+	8 (67%)	13 (87%)	2 (50%)	27 (77%)
Proportion of pre-thrombosis aPL measures that were positive				
<25%	3 (25%)	6 (40%)	0 (0%)	9 (26%)
25-49%	4 (33%)	4 (27%)	1 (25%)	8 (23%)
50-74%	3 (25%)	2 (13%)	1 (25%)	7 (20%)
75%-99%	1 (8%)	2 (13%)	1 (25%)	4 (11%)
100%	1 (8%)	1 (7%)	1 (25%)	7 (20%)
Type of Thrombosis				
Stroke	5 (42%0	4 (27%)	2 (50%)	8 (23%)
Myocardial infarction	1 (8%)	2 (13%)	1 (25%)	6 (17%)
Other arterial thrombosis	4 (33%)	4 (27%)	0	8 (23%)
Digital gangrene	0	1 (7%)	0	1 (3%)
Deep vein thrombosis	2 (17%)	3 (20%)	1 (25%)	9 (26%)
Other venous thrombosis	0	1 (7%)	0	3 (9%)
Number of post-thrombosis aPL Measures				
1-4	1 (8%)	2 (13%)	1 (25%)	4 (11%)
5-9	4 (33%)	0 (0%)	1 (25%)	9 (26%)
10+	7 (58%)	13 (87%)	2 (50%)	22 (63%)
Proportion of post-thrombosis aPL measures that were positive.				
0%	6 (50%)	8 (53%)	0	7 (20%)
<25%	2 (17%)	3 (20%)	1 (25%)	2 (6%)
25-49%	2 (17%)	1 (7%)	0	6 (17%)
50-74%	1 (8%)	2 (13%)	1 (25%)	7 (20%)
75%+	1 (8%)	0 (0%)	1 25%)	8 (23%)
100%	0 (0%)	1 (7%)	1 (25%)(	5 (14%)

[1] This consists of those patients with at least two positive antiphospholipid tests before thrombosis. Some patients can appear in more than one column

Table 2a using the cutoff of 40: Estimated probability of reacquiring antiphospholipid antibody after a thrombosis among those who became negative after the thrombosis

aPL subtype	Number with Thrombosis	Number negative after thrombosis	Number reacquiring a positive result	Estimated risk of reacquiring within 5 years (95% C.I.)
aCL IgG>=40	12	12 (100%)	6 (50%)	44% (21%, 77%)
aCL IgM>=40	15	14 (83%)	6 (43%)	43% (22%, 72%)
aCL IgA>=40	4	3 (75%)	1 (33%)	Too few
Confirmed Lupus Anticoagulant	35	30 (86%)	21 (70%)	76% (57%, 91%)
Pre-thrombosis positive for any of above	45	35 (78%)	24 (69%)	63% (55%, 88%)

Table 2b using the cutoff of 40: Time from thrombosis to first negative antiphospholipid antibody among those who became negative.

Time Since thrombosis	IgG (n=12)	IgM (n=14)	IgA (n=3)	Lupus Anticoagulant (n=30)	Any of the four APLs (n=35)
< 1 year	11 (92%)	14 (100%)	1 (33%)	19 (63%)	25 (71%)
1-3 years	0 (0%)	0 (0%)	1 (33%)	6 (20%)	5 (15%)
>3 years	1 (8%)	0 (0%)	1 (33%)	5 (17%)	5 (15%)
